# Activation of the IGF1R pathway potentially mediates acquired resistance to mutant-selective 3rd-generation EGF receptor tyrosine kinase inhibitors in advanced non-small cell lung cancer

**DOI:** 10.18632/oncotarget.8013

**Published:** 2016-03-09

**Authors:** Ji Hyun Park, Yun Jung Choi, Seon Ye Kim, Jung-Eun Lee, Ki Jung Sung, Sojung Park, Woo Sung Kim, Joon Seon Song, Chang-Min Choi, Young Hoon Sung, Jin Kyung Rho, Jae Cheol Lee

**Affiliations:** ^1^ Department of Oncology, Asan Medical Center, University of Ulsan, College of Medicine, Seoul, Korea; ^2^ Department of Pulmonology and Critical Care Medicine, Asan Medical Center, University of Ulsan, College of Medicine, Seoul, Korea; ^3^ Asan Institute for Life Sciences, Asan Medical Center, University of Ulsan, College of Medicine, Seoul, Korea; ^4^ Department of Pathology, Asan Medical Center, University of Ulsan, College of Medicine, Seoul, Korea; ^5^ Department of Convergence Medicine, Asan Medical Center, University of Ulsan, College of Medicine, Seoul, Korea

**Keywords:** 3rd generation, EGFR-TKI, IGF1R, resistance, NSCLC

## Abstract

Mutant-selective, 3rd-generation EGFR-TKIs were recently developed to control lung cancer cells harboring T790M-mediated resistance. However, the development of resistance to these novel drugs seems inevitable. Thus, we investigated the mechanism of acquired resistance to the mutant-selective EGFR-TKI WZ4002. We established five WZ4002-resistant cells, derived from cells harboring both EGFR and T790M mutations by long-term exposure to increasing doses of WZ4002. Compared with the parental cells, all resistant cells showed 10–100-folds higher resistance to WZ4002, as well as cross-resistance to other mutant-selective inhibitors. Among them, three resistant cells (HCC827/WR, PC-9/WR and H1975/WR) showed dependency on EGFR signaling, but two other cells (PC-9/GR/WR and PC-9/ER/WR) were not. Notably, insulin-like growth factor-1 receptor (IGF1R) was aberrantly activated in PC-9/GR/WR cells in phospho-receptor tyrosine kinase array, consistently accompanied by loss of IGF binding protein-3 (IGFBP3). Down-regulation of IGF1R by shRNA, as well as inhibition of IGF1R activity either by AG-1024 (a small molecule IGF1R inhibitor) or BI 836845 (a monoclonal anti-IGF1/2 blocking antibody), restored the sensitivity to WZ4002 both *in vitro* and xenograft. Taken together, these results suggest that activation of the IGF1R pathway associated with IGFBP3 loss can induce an acquired resistance to the mutant-selective EGFR-TKI, WZ4002. Therefore, a combined therapy of IGF1R inhibitors and mutant-selective EGFR-TKIs might be a viable treatment strategy for overcoming acquired resistance.

## INTRODUCTION

During the last decade, epidermal growth factor receptor (EGFR) tyrosine kinase inhibitors (TKIs) established a remarkable therapeutic benefit in the patients with advanced non small cell lung cancer (NSCLC) harboring EGFR activating mutations [1–7]. Unfortunately, however, 1st-generation EGFR-TKIs such as gefitinib and erlotinib are ultimately limited by the inevitable development of acquired resistance after a median of 10 to 12 months [8–11]. T790M is the most common mechanism of acquired resistance observed in approximately 50% to 60% of patients. In this gatekeeper mutation, a well-conserved threonine at codon 790 in exon 20 of EGFR undergoes substitution to a bulkier methionine, which leads to steric hindrance of erlotinib binding in the ATP-kinase-binding pocket [8]. Although 2nd-generation EGFR-TKIs including afatinib (BIBW2992) and dacomitinib (PF299804) effectively inhibited T790M-containing cell lines in several preclinical models at high concentration and showed modest clinical potency, they eventually failed to prove universal clinical benefit [12–16]. This unmet need finally led to the development of mutant-selective, 3rd-generation EGFR TKIs, which comprises the irreversible pyrimidine-based WZ4002 and newer compounds of AZD9291, CO-1686, and HM61713 [17]. Strikingly, recent preclinical and preliminary clinical data demonstrated an outstanding clinical efficacy of 3rd-generation EGFR-TKIs in patients with advanced NSCLC harboring T790M [18–23]. However, in the real world practice, patients ultimately experience disease progression regardless of the favorable clinical responses of 3-generation EGFR TKIs. Thus, considering this successive development of acquired resistance beyond T790M, monotherapy with 3rd-generation EGFR-TKIs would be insufficient to control the disease. Facing these concerns, efforts to discover the mechanisms of novel acquired resistance have been increasingly made in recent studies, which addressed insulin-like growth factor receptor (IGF1R) [24] and extracellular signal-regulated kinase (ERK) signaling [25] as potential mediators of acquired resistance to WZ4002. These investigations also suggest the significance of combination treatment strategies in the backbone of EGFR-TKIs to more efficiently manage the acquired resistance, and further prevent and predict the emergence of novel resistance [24–27].

In this context, current study is to evaluate whether the IGF1R signaling pathway contributes to the emergence of acquired resistance to the mutant-selective EGFR-TKI WZ4002, using both BI 836845, a selective anti-IGF1/2 monoclonal antibody and AG-1024, a small-molecule chemical inhibitor. Accordingly, five cell-lines resistant to WZ4002 were constructed including PC-9/GR/WR and PC-9/ER/WR cell lines, which were concurrently resistant clones to both 1st- and 3rd-generation EGFR-TKIs.

## RESULTS

### Mutant-selective EGFR-TKIs can overcome the resistance regardless of T790M allele frequency

**Figure 1:**
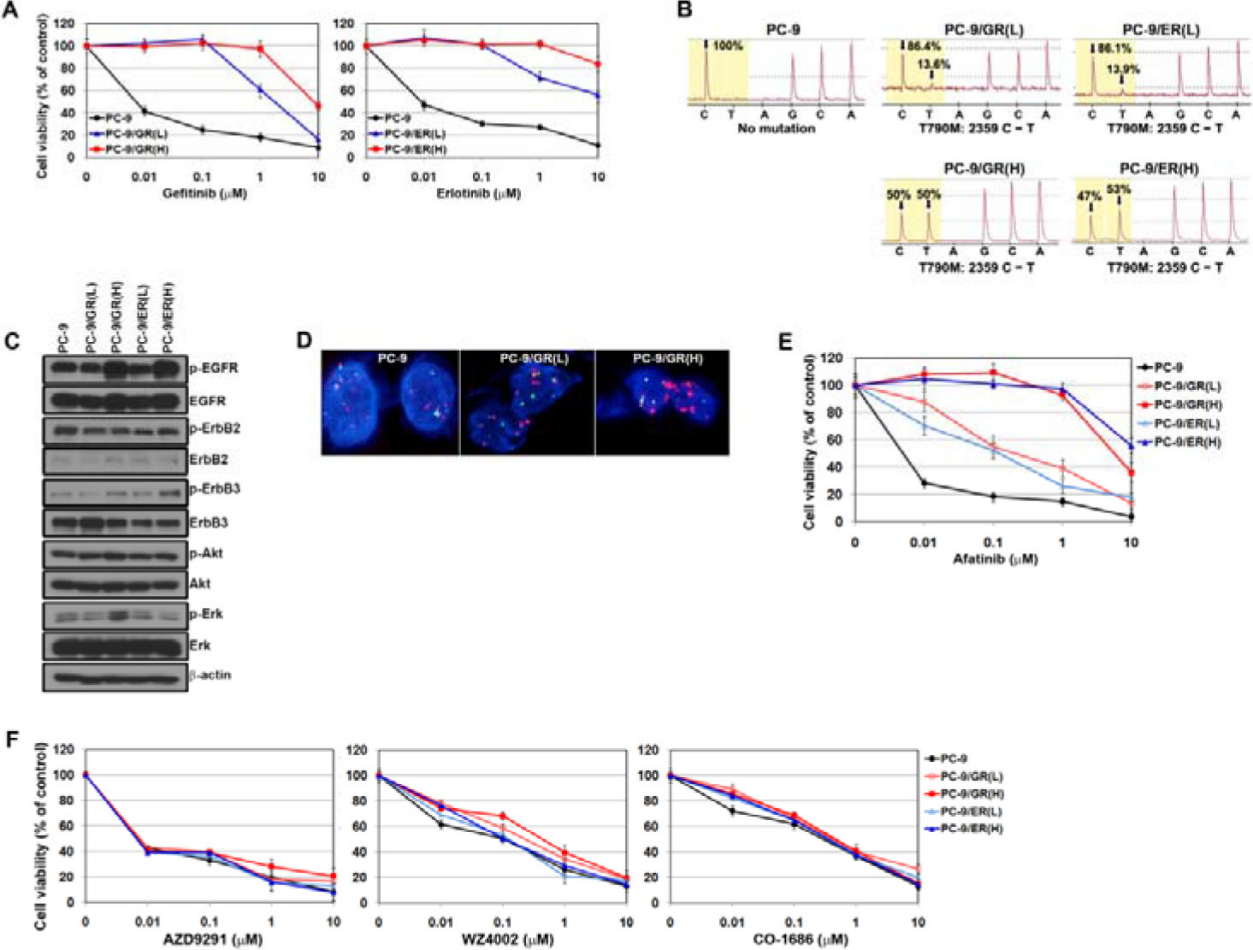
Mutant-selective EGFR-TKIs can overcome gefitinib or erlotinib resistance caused by T790M (**A**) Cells were treated with gefitinib or erlotinib, and then the sensitivity to drugs was determined by MTT assay. (**B**) Pyrosequencing of EGFR-TK exon 20 revealed a C-to-T base pair change (arrows) corresponding to T790M. (**C**) The basal expressions of EGFR-related proteins in parental and resistant cells were determined by Western blotting. (**D**) The EGFR gene copy number was determined by fluorescent *in situ* hybridization (FISH). EGFR gene amplification was detected by FISH using probe against centromere of chromosome 7 (CEP7, green) and EGFR (red). Nuclei (blue) were counterstained with DAPI. (**E** and **F**) Cells were treated with various EGFR-TKIs, and then the sensitivity to drugs was determined by MTT assay.

Gefitinib- and erlotinib-resistant cells harboring the T790M mutation were established as in a previous study [28]. These cells were continuously exposed to each drug for 6 months, and were finally more resistant to each drug than the PC-9/GR (L) and PC-9/ER (L) cells (Figure 1A). Accordingly, they showed enhanced T790M allele frequency (from 13.6% to 50% in gefitinib-resistant cells and from 13.9% to 53% in erlotinib-resistant cells; Figure 1B). The levels of EGFR phosphorylation were markedly increased in PC-9/GR (H) and PC-9/ER (H) cells, whereas there was only a modest difference in total EGFR (Figure 1C). Using FISH, we also observed increased amplification of EGFR genes in PC-9/GR (H) cells compared with PC-9 or PC-9/GR (L) cells (Figure 1D). Taken together, these findings indicate that the increase in T790M allele frequency by gene amplification leads to more potent resistance to EGFR-TKIs.

Previous studies suggested that 2nd-generation EGFR-TKIs (irreversible TKIs: neratinib, afatinib and dacomitinib) were able to effectively inhibit EGFR and EGFR-related downstream molecules as well as cell proliferation in EGFR-mutant cells with the T790M mutation [29]. As all resistant cells were still EGFR dependent (data not shown), we evaluated the efficacy of afatinib in these resistant cells. As shown in Figure 1E, cells with high frequency of the T790M allele were more resistant to afatinib than cells with low frequency. However, the cells were still sensitive to 3rd-generation EGFR-TKIs (mutant-selective EGFR-TKIs), regardless of the frequency of T790M allele (Figure 1F).

### WZ4002-resistant cells showed the cross-resistance to other tyrosine kinase inhibitors

To investigate the mechanisms of acquired resistance to WZ4002, we established the resistant cells in EGFR-mutant cells with or without the T790M mutation. As indicated in Table 1, all WZ4002-resistant cells acquired approximately 10- to 100-folds higher 50% inhibitory dose (IC_50_) to WZ4002 than each parental cell (at the IC_50_: above 100-fold in HCC827/WR, H1975/WR, PC-9/GR/WR and PC-9/ER/WR; above 10-fold in PC-9/WR). Furthermore, all WZ4002-resistant cells showed cross-resistance to other EGFR-TKIs, including 1st-, 2nd- or 3rd-generation EGFR-TKIs.

**Table 1:** Generation of acquired resistance to WZ4002 in NSCLC cells

Cell lines	IC_50_ values (μM, mean ± S.D.)					
	Gefitinip	Erlotinip	Afatinip	AZD9291	WZ4002	CO-1686
**HCC827**	0.07 (± 0.03)	0.06 (± 0.02)	0.04 (± 0.03)	< 0.01	0.1 (± 0.05)	0.12 (± 0.08)
**HCC827/WR**	> 10	> 10	> 10	> 10	> 10	> 10
**H1975**	7.5 (± 1.5)	8.2 (± 0.9)	0.08 (± 0.02)	0.05 (± 0.04)	0.12 (± 0.05)	0.8 (± 0.1)
**H1975/WR**	> 10	> 10	> 10	> 10	> 10	> 10
**PC-9**	0.08 (± 0.02)	0.07 (± 0.02)	< 0.01	< 0.01	0.3 (± 0.2)	0.4 (± 0.3)
**PC-9/WR**	7.5 (± 1.3)	> 10	6.9 (± 2.2)	2.4 (± 1.5)	8.2 (± 2.2)	> 10
**PC-9/GR**	6 (± 1.2)	> 10	0.7 (± 0.3)	< 0.01	0.4 (± 0.2)	0.7 (± 0.4)
**PC-9/GR/WR**	> 10	> 10	> 10	7.2 (± 1.4)	> 10	> 10
**PC-9/ER**	5.5 (± 2.1)	8.8 (± 3.2)	0.6 (± 0.1)	< 0.01	0.3 (± 0.1)	0.6 (± 0.1)
**PC-9/ER/WR**	> 10	> 10	> 10	> 10	> 10	> 10

**Figure 2:**
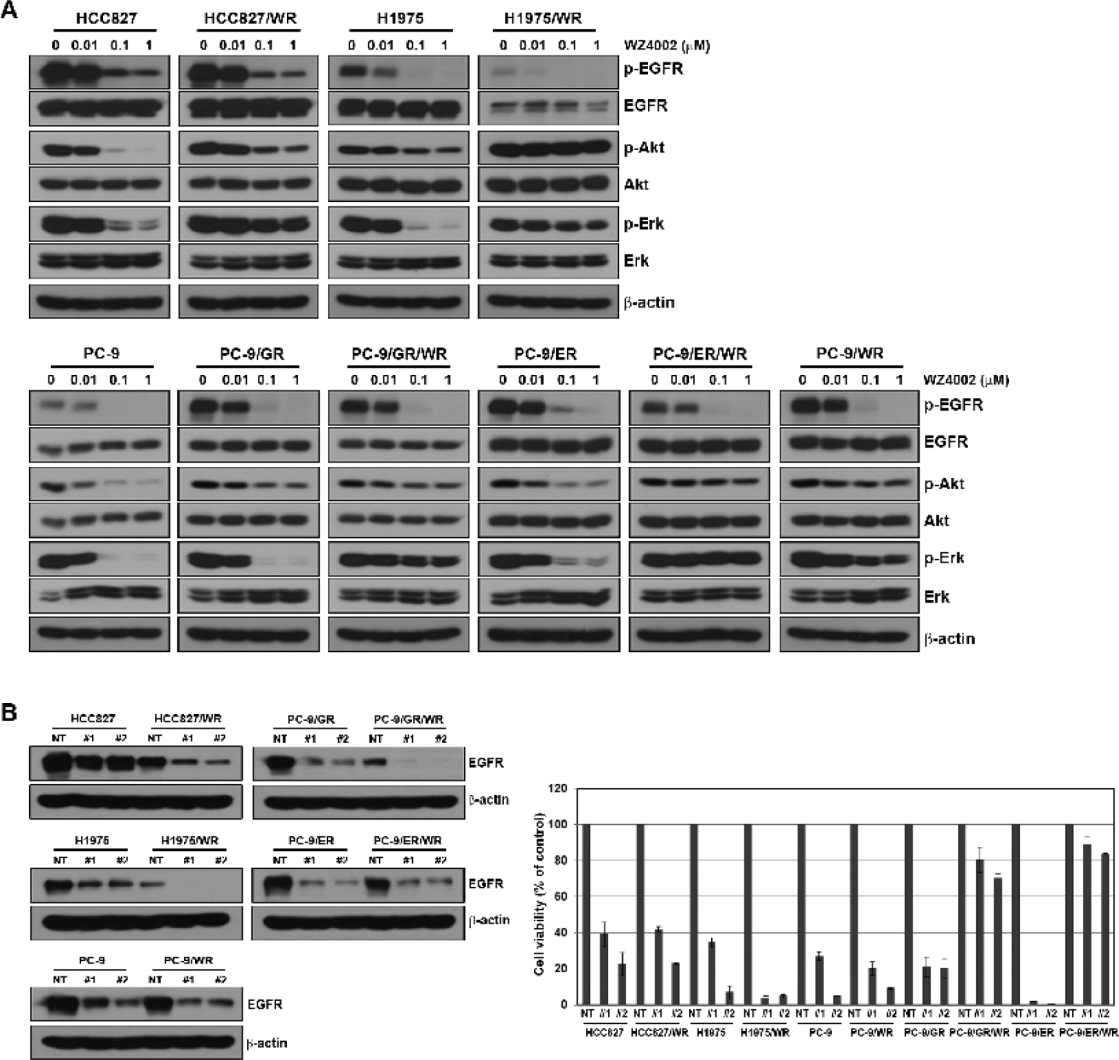
Comparison of dependency on EGFR signaling in cells with acquired resistance to WZ4002 (**A**) Cells were treated with or without the indicated doses of WZ4002 for 5 h. EGFR-related signal molecules were assessed using Western blot analysis. (**B**) Lentiviral constructs containing negative control (NT) and EGFR shRNAs were infected into parental or resistant cells, and EGFR suppression was confirmed by Western blot analysis. Cell viability was measured by cell counting.

We examined the changes in EGFR-related molecules in the presence of WZ4002. WZ4002 treatment reduced the levels of phosphorylated EGFR in both parental and resistant cells, but the levels of phosphorylated Akt and Erk were not decreased in all WZ4002-resistant cells (Figure 2A). To further determine if WZ4002-resistant cells depend on EGFR signaling for growth, we silenced the EGFR gene by lenti-viral infection of EGFR shRNA. Two different EGFR-specific shRNAs substantially decreased the amount of EGFR, and it determined by Western blotting (Figure 2B). Interestingly, three WZ4002-resistant cells (HCC827/WR, H1975/WR and PC-9/WR) showed EGFR dependency for cell growth, whereas two WZ4002-resistant cells (PC-9/GR/WR and PC-9/ER/WR) were independent of EGFR signaling.

### Activation of IGF1R was associated with acquired resistance to WZ4002

**Figure 3:**
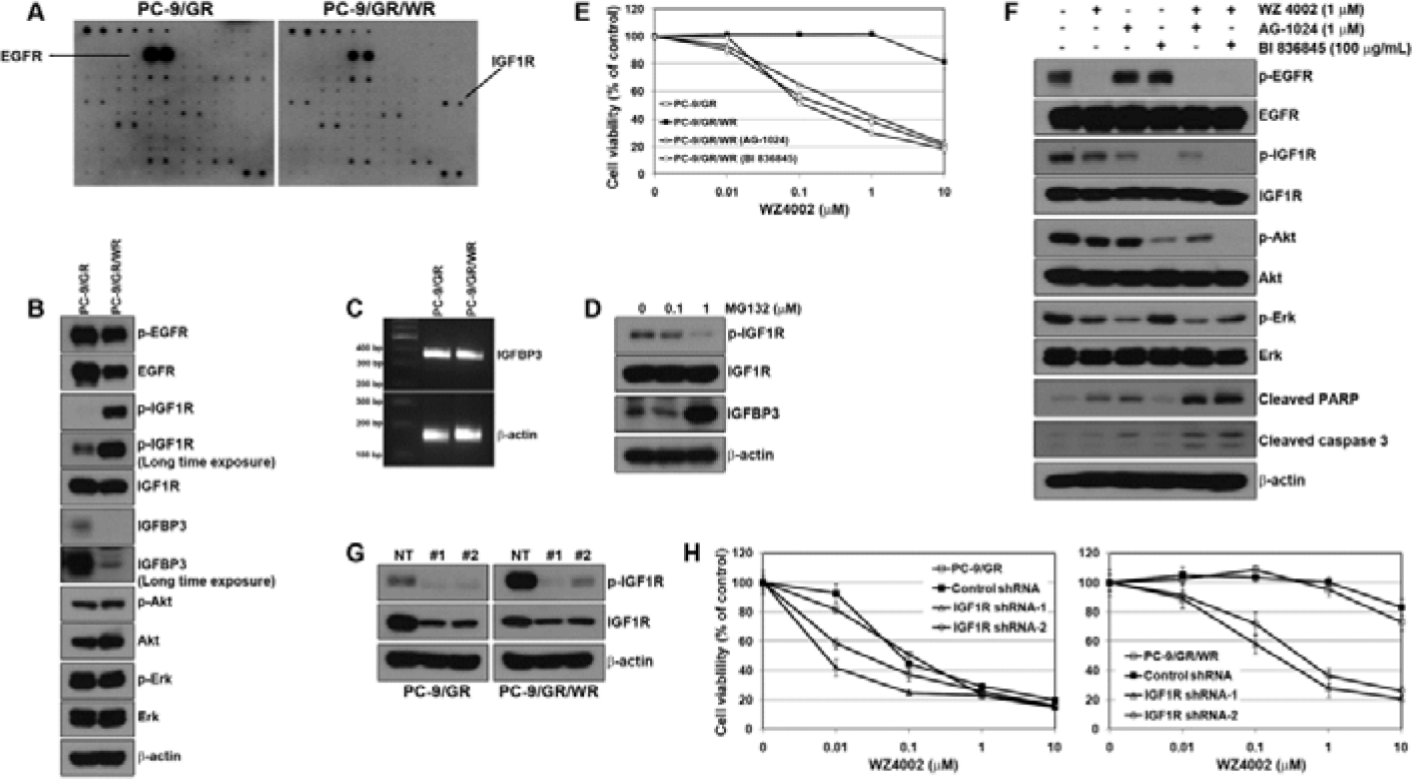
Activation of IGF1R was associated with the resistance to WZ4002 (**A**) Cells were grown to confluence, and then cell lysates were prepared by protein extraction. Phospho-receptor tyrosine kinase array was performed as described in Materials and Methods. (**B**) EGFR and IGF1R-related signal molecules in basal level were assessed using Western blot analysis. (**C**) IGFBP3 mRNA was determined by RT-PCR. (**D**) PC-9/GR/WR cells were treated with MG132 for 6 h. Restored IGFBP3 was determined by Western blot analysis. (**E**) Cells were treated with WZ4002, AG-1024, BI 836845, or a combination of WZ4002 with one of the other 2 drugs for 72 h. Cell viability was measured by MTT assay. (**F**) PC-9/GR/WR cells were treated with drugs as in (E). After 48 h, cells were harvested and subjected to Western blotting using the indicated antibodies. (**G** and **H**) Lentiviral constructs containing negative control (NT) and IGFR shRNAs were infected into PC-9/GR or PC-9/GR/WR cells, and IGFR silencing was confirmed by Western blot analysis (G). After the selection of puromycin, cells were treated with the indicated doses of WZ4002, and then cell viability was determined by MTT assay (H).

To identify other bypass signals contributing to the resistance, we performed a multi-RTK array. Interestingly, the activation of IGF1R was detected in PC-9/GR/WR cells, whereas no change was detected in the other four WZ4002-resistant cells (Figure 3A). To confirm whether the activation of IGF1R was associated with the acquired resistance to WZ4002, the basal levels of IGF1R activity were determined by Western blotting. Consistent with the RTK array, IGF1R activity was found to be dramatically increased in PC-9/GR/WR cells (Figure 3B). Previous studies already suggested that resistance to EGFR-TKIs was mediated by the activation of IGF1R pathway due to the reduction of IGFBP3 expression via promoter methylation [24, 30]. Although the reduction in IGFBP3 was observed in PC-9/GR/WR cell, both parental and resistant cells showed similar levels of IGFBP3 mRNA (Figure 3C). In addition, treating 5-aza-2′-deoxycytidine (5-aza-dC) did not significantly increase the levels of IGFBP3 protein in PC-9/GR/WR cells (Supplementary Figure S1). However, treatment with MG132, a well-known proteasome inhibitor, recovered the levels of IGFBP3 protein (Figure 3D) suggesting that down-regulation of IGFBP3 might also occur at the post-transcriptional level.

To investigate whether inhibition of IGF1R could overcome the acquired resistance to WZ4002 in PC-9/GR/WR cell, we used a small-molecule, chemical inhibitor of IGF1R (AG-1024) and a ligand-neutralizing monoclonal antibody (BI 836845) as well as shRNA against IGF1R. Either AG-1024 or BI 836845 alone failed to demonstrate cytotoxic effects on both parental and resistant cells until their concentrations reached 1 μM and 100 μg/mL, respectively (Supplementary Figure S2). However, combined treatment of WZ4002 either with AG-1024 or BI 836845 led to recovery of sensitivity to WZ4002 in PC-9/GR/WR cells (Figure 3E). As expected, the combination of WZ4002 with AG-1024 or BI 836845 completely suppressed the activity of down-stream EGFR-related signaling molecules, and further induced apoptotic proteins such as PARP and caspase-3 (Figure 3F). In addition, both parental and resistant cells were infected with two different IGF1R-specific shRNA lentiviral particles, which substantially suppressed the amount and activity of IGF1R in Western blotting (Figure 3G). As a consequence, responsiveness to WZ4002 was restored in resistant cells through silencing of IGF1R (Figure 3H). Interestingly, parental cells also showed a slight recovery of sensitivity to WZ4002. Taken together, the genetic and pharmacological data showed that IGF1R could be required for the acquisition of resistance to WZ4002.

### Inhibition of IGF1R activity overcame acquired resistance to WZ4002 in PC-9/GR/WR tumor xenograft *in vivo*

**Figure 4:**
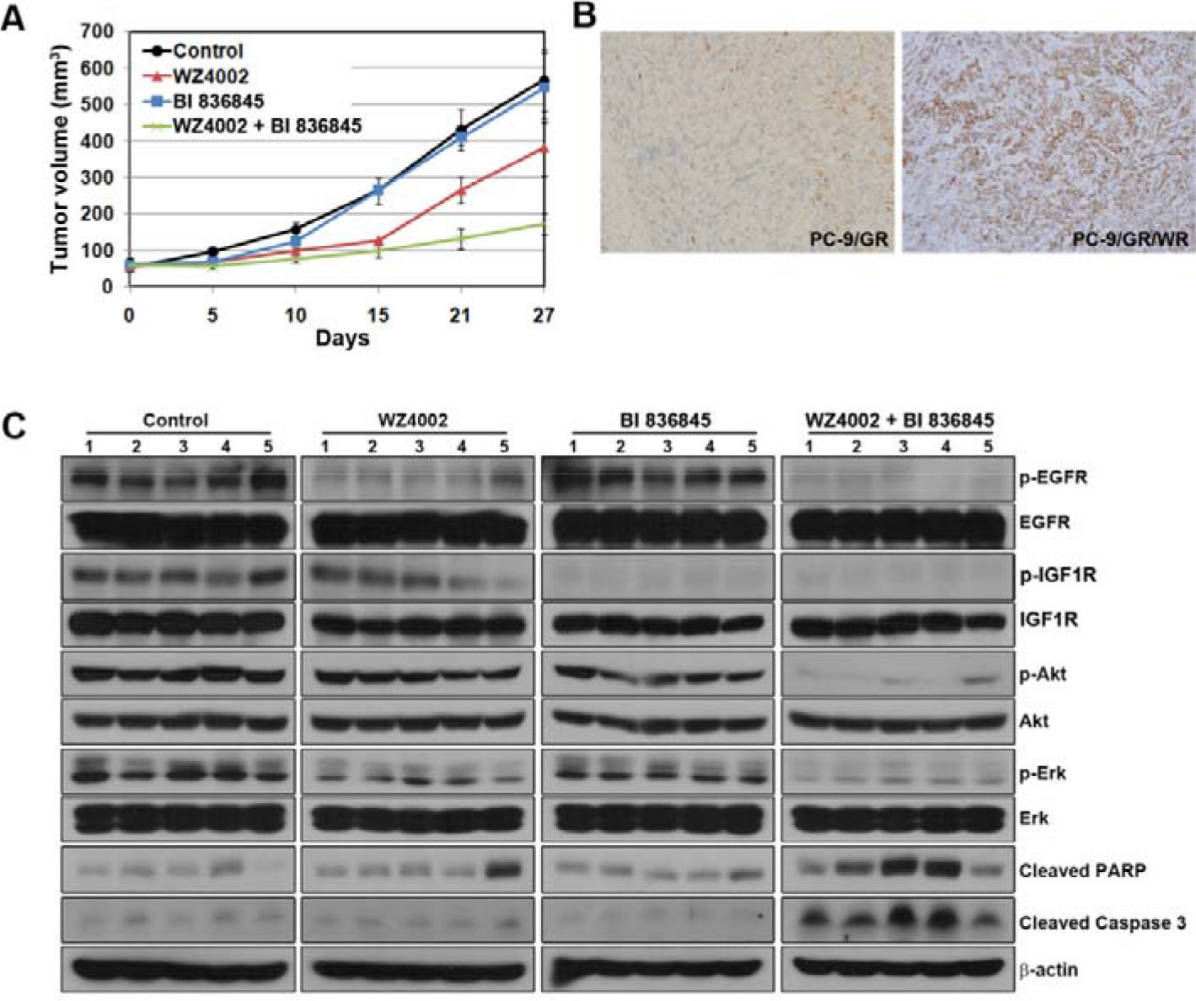
Addition of BI 836845 to WZ4002 overcomes acquired resistance to WZ4002 in a xenograft model (**A**) SCID mice bearing established PC-9/GR/WR tumor cell xenografts were treated with each drug as described in Materials and Methods. The length and width of the tumors were measured at the days indicated and tumor volumes were calculated. The bars represent mean tumor volume **±** S D. (**B**) Evaluation of phosphorylated IGF1R was measured by IHC in tumors from xenografts. (**C**) Tumors from each group were homogenized for lysate preparation and analyzed by Western blotting.

To further evaluate the efficacy of the combination of BI 836845 with WZ4002, PC-9/GR/WR tumor xenografts were established in SCID mice. WZ4002 was administered orally 5 days a week and BI 836845 was given intraperitoneally 2 times a week at the indicated times (Figure 4A). Monotherapy with each drug either showed only a slight decrease or comparable growth compared with control group in tumor growth, whereas combined treatment resulted in substantial growth inhibition (Figure 4A). We then compared the changes in EGFR-related down-stream molecules in xenografts treated with each regimen. IGF1R activation was found in tumors from the PC-9/GR/WR xenograft (Figure 4B). Consistent with the *in vitro* observations, the activities of EGFR, IGF1R, Akt and Erk were completely inhibited only by the combined therapy (Figure 4C). In addition, apoptosis was only induced by the combination of WZ4002 and BI 836845. Coupled with the *in-vitro* data, these results altogether suggest that activation of IGF1R signaling might be significantly correlated with acquired resistance to WZ4002.

## DISCUSSION

Third generation EGFR-TKIs have emerged as the treatment-of-choice for EGFR mutant, advanced NSCLC in recent years, particularly in patients who develop T790M mutation after showing a response to prior EGFR-TKIs such as erlotinib or gefitinib. However, it ultimately cannot escape from the hurdle of evolving acquired resistance. Therefore, the unmet needs currently lie in the identification of the potential signaling pathways involved in development of novel acquired resistance. In our study, we established two resistant cells (PC-9/GR and PC-9/ER) harboring T790M, as in a previous study [31]. These cells were cross-resistant to the 1st-generation EGFR-TKIs gefitinib and erlotinib but sensitive to the 2nd-generation EGFR-TKI afatinib. Further exposure of these cells to 1st-generation EGFR-TKIs led them insensitive to afatinib as well as gaining higher resistance to 1st-generation EGFR-TKIs. However, they were still sensitive to 3rd-generation EGFR-TKIs. These cells presented a high frequency of T790M through amplification of the T790M allele, which was consistent with previous studies [32–34]. It suggests that the frequency of the T790M allele might be associated with sensitivity to 2nd generation EGFR-TKIs, but not with 3rd generation EGFR-TKIs. In addition, we developed five WZ4002-resistant cell lines using EGFR-mutant cell lines (HCC827 and PC-9), gefitinib or erlotinib-resistant cells and H1975 cells harboring T790M. None of the WZ4002-resistant cells had new mutations such as C797S (data not shown). Among them, three WZ4002-resistant cells (HCC827/WR, H1975/WR, and PC-9/WR) showed dependency on EGFR, whereas two WZ4002-resistant cells (PC-9/GR/WR and PC-9/ER/WR) no longer depended on EGFR signaling. These suggest the activation of a bypass signaling pathway which enables survival of cells in the absence of EGFR signaling. As shown in Figure 3, PC-9/GR/WR had aberrantly activated IGF1R signaling whereas phosphorylated EGFR was adequately inhibited by WZ4002. More importantly, reversal of resistance with a combination of WZ4002 and an antibody binding to IGF1/2, BI 836845, reinforces the significance of the activated IGF1R signaling pathway as a potential mechanism of acquired resistance. The potency of combination therapy with 3rd-generation EGFR-TKIs and IGF1R signaling inhibitors was also suggested. However, unlike the situation in PC-9/GR/WR cells, we could not find any activated bypass signaling pathway in PC-9/ER/WR cells, probably because of the limited coverage of the RTK array kit. Interestingly, T790M loss was detected only in PC-9/ER/WR cells by sequencing (data not shown). According to a recent study by Thress et al. [35, 36], 4 of 15 patients treated with AZD9291 were found to have T790M mutation loss without any identified resistance mechanism. The PC-9/ER/WR cells in our study seem to be well matched with these cases, and we should continue to define the exact mechanism of resistance in PC-9/ER/WR cells.

Although some earlier studies have suggested that IGF1R activation is associated with acquired resistance to EGFR-TKIs in cells with wild-type or mutant-type EGFR [24, 30, 37, 38], our study is the first to provide evidence that IGF1R activation might be associated with acquired resistance to 3rd-generation EGFR-TKIs that develops after failure of prior treatment with 1st-generation EGFR-TKIs (gefitinib and erlotinib). This dual resistant condition has particular significance in the clinical context, given that most EGFR-mutant patients with advanced NSCLC are usually treated with 1st-generation EGFR-TKIs in the first-line setting and subsequently progress. Even with the recently highlighted advantages of 1st-line 3rd-generation EGFR-TKIs regarding delayed emergence of acquired resistance as well as cumulative toxicities from sequential EGFR-TKIs treatment, its therapeutic benefit remains unclear, and comparative trials evaluating its feasibility are still ongoing. Notably, in our study, compared with PC-9/GR and PC-9/ER cells, similar times were required to create the HCC827/WR and PC-9/WR cells, suggesting that 1st exposure to WZ4002 could not delay the acquisition of de novo resistance to WZ4002. More importantly, HCC827/WR and PC-9/WR cells showed cross-resistance to all generations of EGFR-TKIs, which precludes further treatment options in the current practice. Based on these data, the potential pitfalls of optimism in 1st-line treatment with 3rd-generation EGFR-TKIs should not be overlooked. In a previous study by Cortot et al. [24], activation of the IGF1R pathway with IGFBP3 loss was suggested to be involved in resistance to irreversible EGFR-TKIs such as WZ4002 or PF299804. Herein, BMS536924, an IGF1R inhibitor, showed its efficacy in combination with WZ4002 or PF299804. However, neither WZ4002- nor PF299804-resistant cell lines harbored T790M, excluding prior treatment with 1st-generation inhibitors. Synergism of combined IGF1R (NVP-AEW541) and EGFR inhibitors was also previously reported, but that study involved EGFR wild-type and erlotinib-resistant cell lines [37]. In this point of view, current findings seem to better fit real world practice.

IGFBP, the main carrier protein for IGFs, was first investigated with the EGFR wild-type A431 cell line resistant to gefitinib [30]. Recently, loss of IGFBP3 has been increasingly reported to induce activation of IGF1R signaling and resistance to EGFR-TKIs, and the addition of recombinant IGFBP3 was sufficient to recover sensitivity to 3rd-generation EGFR-TKIs [24]. Of note, in our study, loss of IGFBP3 was associated with a proteolytic process rather than hypermethylation of promoter during the transcription. It was supported by the increased level of IGFBP3 after the application of proteasome inhibitor. This salient finding suggests the possibility of IGFBP3 loss at the post-transcriptional level. Activation of the mitogen-activated protein kinase (MAPK) pathway is regarded as another significant potential resistance mechanism. Aberrant amplification of extracellular signal-regulated kinase (ERK) 2 gene by activation of MAPK1 or by suppression of its negative regulators was observed in WZ-resistant, T790M positive cell lines. Thus, the addition of MAP-ERK (MEK) or Erk inhibitor to WZ4002 dramatically restored sensitivity to WZ4002 [25]. However, in the current study, we did not observe any signaling change in the MAPK pathway. Accordingly, combination treatment with WZ4002 and U0126, an Erk inhibitor, was insufficient to overcome the resistance in all WZ4002-resistant cells (Supplementary Figure S3).

There are several limitations to our study. First, IGF1R activation was limitedly found in PC-9/GR/WR cells among various resistant cell lines. Interestingly, it was not even observed in PC9/ER/WR cells, which are regarded to reflect the same clinical context as PC-9/GR/WR cells. Although we cannot accurately reason these results, it seems partly explainable in the background of the molecular heterogeneity of tumor clones, as we remarked when discussing our discordant results of T790M loss between these two cells. Unpredictable biochemical differences, even among the same generation of EGFR-TKIs, should also be considered. In the context, although we determined the cross-resistance of PC-9/GR/WR and PC-9/ER/WR cells to other 3rd-generation EGFR-TKIs, IGF1R activation further needs to be reproducible in resistant clones using other 3rd-generation EGFR-TKIs.

In conclusion, current study suggests that activation of the IGF1R signaling pathway is significantly associated with the acquisition of resistance to 3rd-generation EGFR-TKIs, which is accompanied by loss of IGFBP3. Given that 3rd-generation EGFR-TKIs will be more widely used in real world practice as the key treatment for advanced NSCLC in the near future, a combination therapy with 3rd-generation EGFR-TKIs and IGF1R signaling inhibitor might be an attractive treatment option for the cautious optimism toward novel EGFR-TKIs. Actual incidence of its aberrancy should be further clarified in patient samples to maximize the efficacy of current combination therapy.

## MATERIALS AND METHODS

### Cell culture and reagents

The human NSCLC cell lines (HCC827 and H1975) were obtained from the American Type Culture Collection (Rockville, MD). PC-9 cells were a gift from F. Koizumi and K. Nishio (National Cancer Center Hospital, Tokyo, Japan). Cells were cultured in RPMI 1640 medium containing 10% FBS, 2 mmol/L L-glutamine, and 100 units/mL of penicillin and streptomycin, and maintained at 37°C in a humidified chamber containing 5% CO_2_. Gefitinib, erlotinib, afatinib, AZD9291, CO-1686, WZ4002, AG-1024 and MG132 were purchased from Selleck Chemicals (Houston, TX). BI836845 was kindly provided by Boehringer Ingelheim (Vienna, Austria).

### Generation of WZ4002-resistant cells

PC-9/GR (L), and PC-9/ER (L) cells were established as in a previous study [31]. These cells were additionally cultured under the continuous stress of gefitinib or erlotinib treatment for 6 months. These resistant sublines were designated PC-9/GR (H), and PC-9/ER (H) cells, respectively. To create WZ4002-resistant cell lines, NSCLC cells were exposed to increasing concentrations of WZ4002, similar to previous studies [31, 39]. WZ4002-resistant cells are referred to as cell name/WR (i.e., HCC827/WR, PC-9/WR, H1975/WR, PC-9/GR/WR and PC-9/ER/WR).

### MTT assay

Cells (5 × 10^3^) were seeded in 96-well sterile plastic plates overnight and then treated with the relevant agents. After 72 h, 15 μL of MTT solution (5 mg/mL) was added to each well and the plates were incubated for 4 h. Crystalline formazan was solubilized with 100 μL of a 10% (w/v) SDS solution for 24 h, and then absorbance at 595 nm was read spectrophotometrically using a microplate reader. The results are representative of at least three, independent experiments, and the error bars signify standard deviations (SDs).

### Pyrosequencing assay for the T790M mutation

The PCR amplification of exon 20 EGFR was performed as previously described [28]. PCR products were sequenced using the Pyrosequencing PSQ96 HS System (Biotage, Charlotte, NC) according to the manufacturer's instructions.

### Fluorescent *in situ* hybridization

Fluorescent *in situ* hybridization (FISH) was performed using an EGFR/CEP7 probe according to the manufacturer's protocol (Abbott Molecular, Abbott Park, IL). Briefly, the fixed slides were incubated in 2 × NaCl-sodium citrate buffer (SSC; pH 7.0) at 75°C for 20 min, digested with proteinase K at 37°C for 60 min, rinsed in 2 × SSC at room temperature for 5 min, co-denatured at 85°C for 15 min, hybridized at 37°C for 16 h and washed with 2 × SSC containing 0/3% NP-40. Nuclei were counterstained with 4′,6-diaminino-2-phenylindole (DAPI, Abbott Molecular). FISH signals for each locus-specific FISH probe were analyzed using an epifluorescence microscope with a single interference filter set for green (FITC), red (Texas red) and blue (DAPI), as well as dual (red/green) and triple (blue, red, green) band pass filters.

### Western blot analysis

Cells were lysed in buffer containing 137 mmol/L NaCl, 15 mmol/L EGTA, 0.1 mmol/L sodium orthovanadate, 15 mmol/L MgCl_2_, 0.1% Triton X-100, 25 mmol/L MOPS, 100 mmol/L phenylmethylsulfonyl fluoride, and 20 mmol/L leupeptin, adjusted to pH 7.2. Lysis of tumor specimens was performed using Omni Tissue Homogenizer (TH; Omni International, Kennesaw, GA). Antibodies specific for p-EGFR (Tyr1173), EGFR, Akt, Erk, IGFBP3, IGF1R and actin were obtained from Santa Cruz Biotechnology (Santa Cruz, CA), and antibodies for p-ErbB2 (Tyr1221/1222), ErbB2, p-ErbB3 (Tyr1289), ErbB3, p-IGF1R (Tyr1135/1136), p-Akt (Ser473), p-Erk (Thr202/Tyr204), caspase-3 and PARP-1 were purchased from Cell Signaling Technology (Beverly, MA). Proteins were detected with an enhanced chemiluminescence Western blotting kit (Amersham Biosciences) according to the manufacturer's instructions.

### Lentiviral infection

EGFR and IGF1R shRNA lentiviral particles were purchased from Sigma-Aldrich (St. Louis, MO). For lentivirus infection, cells were infected with shGFP, shEGFR or shIGF1R lentivirus. To validate EGFR dependency, cells were infected with shGFR or shEGFR for 48 h, and then treated with 2 μg/mL puromycin for 48 h. The viability (living and dead cells) of cells was determined using an ADAM-MC automatic cell counter (NanoEnTek) according to the manufacturer's instructions. For MTT assay, cells were infected with shGFR or shIGF1R. MTT assay was performed after selection of 2 μg/mL puromycin.

### Reverse transcription-PCR

To evaluate the mRNA levels of IGFBP3, reverse transcription (RT)-PCR of IGFBP3 was performed as previously described [40].

### Phospho-receptor tyrosine kinase array analysis

Phospho-receptor tyrosine kinase (RTK) array analysis was performed according to the manufacturer's instruction (RayBiotech, Norcross, GA). Experiments were performed as previously described [41].

### Xenograft studies

To establish the xenograft model, female severe combined immunodeficiency (SCID) mice (18–20 g, 6 weeks of age) were purchased from Charles River Laboratories. All experimental procedures were conducted following a protocol approved by the Institutional Animal Care and Use Committee of Asan Institute for Life Sciences (2015-02-062). Tumors were grown by implanting cells (1–5 × 10^6^ cells/0.1 mL) in 50% Matrigel (BD Biosciences), and subcutaneously injected into the right flank of the animals. Drug treatment was started in 5 mice per group when the tumor volume reached 50–100 mm^3^ with control (10% 1-methyl-2-pyrrolidinone: 90% PEG-300, oral gavage), WZ4002 (30 mg/kg, oral gavage, 5 days a week), BI836845 (100 mg/kg, intraperitoneal, 2 days a week), or WZ4002 plus BI836845. To estimate the tumor size, the length (*L*) and width (*W*) of each tumor were measured using calipers, and the tumor volume (TV) was calculated as TV = (*L*× *W*^2^)/2. To evaluate the p-IGF1R in tumors from xeongrafts, p-IGF1R was measured by immunohistochemistry (IHC) with p-IGF1R antibody (phosphor Y1161, Abcam). IHC staining was performed using the EnVision Plus staining kit (DakoCytomation).

## SUPPLEMENTARY MATERIALS FIGURES


