# Supplementary material for: Activation of the IGF1R pathway potentially mediates acquired resistance to mutant-selective 3rd-generation EGF receptor tyrosine kinase inhibitors in advanced non-small cell lung cancer
Source: Oncotarget. 2016 Mar 9;7(16):22005–15. doi: 10.18632/oncotarget.8013 (PMC5008340; doi:10.18632/oncotarget.8013)
Supplement: Supplementary file 1 [file oncotarget-07-22005-s001.pdf]

Activation of the IGF1R pathway potentially mediates acquired resistance to mutant-selective 3rd-generation EGF receptor tyrosine kinase inhibitors in advanced non-small cell lung cancer

Supplementary Materials

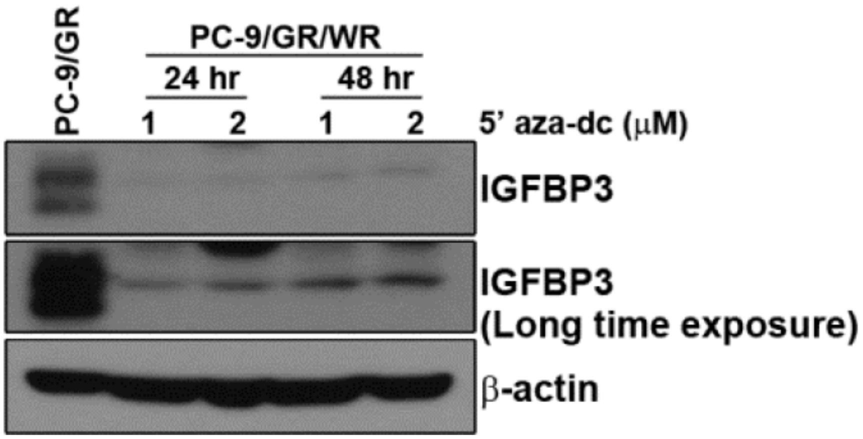

Supplemenatry Figure S1: Effect of 5-aza-dC treatment on IGFBP3 expression. Cells were treated with the indicated time or dose of 5-aza-dC. IGFBP3 expression was determined by Western blotting.

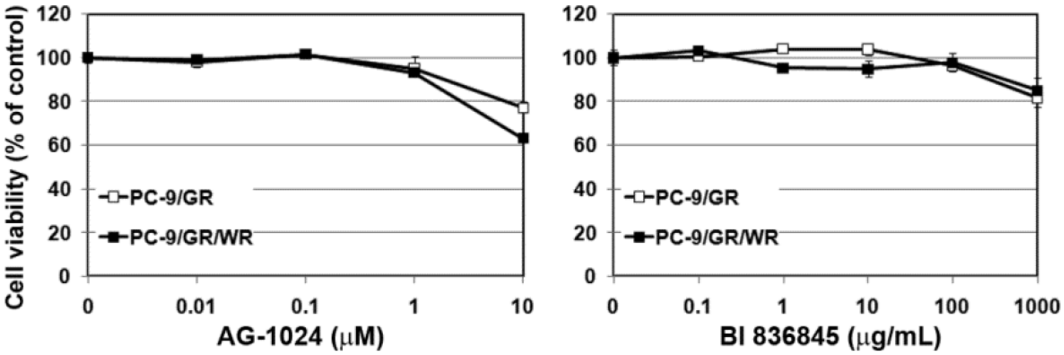

Supplemenatry Figure S2: Cytotoxicity of AG-1024 or BI 836845. Cells were treated with the indicated dose of AG-1024 or BI 836845 for 72 h. Cellular viability was determined by MTT assay.

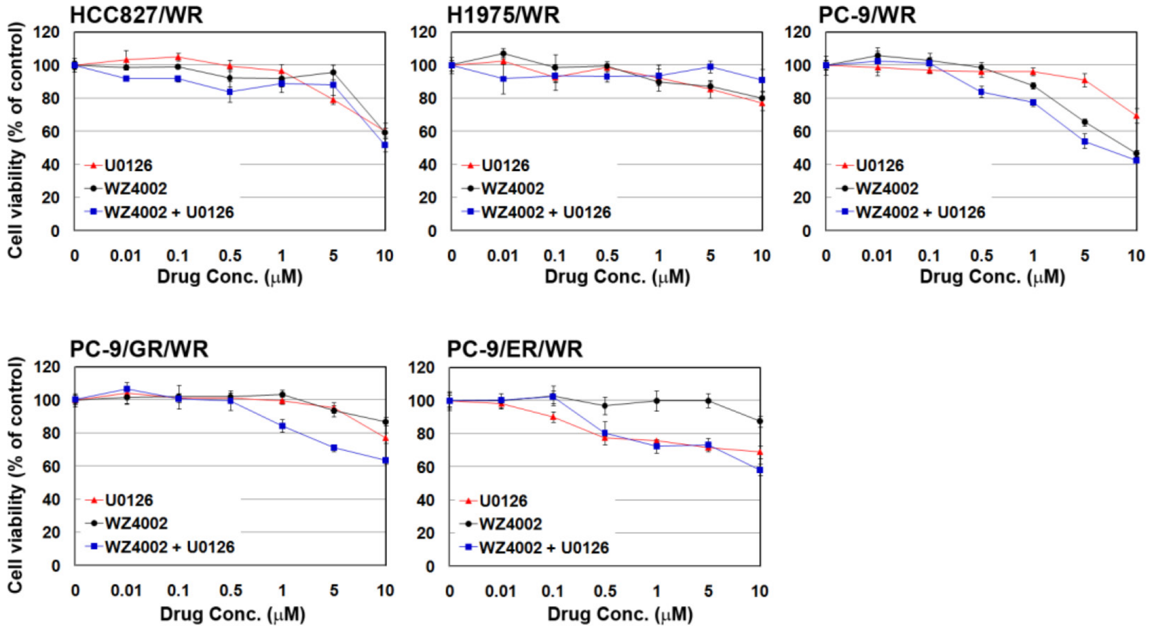

Supplemenatry Figure S3: Effect of combination WZ4002 and U0126 in cells with acquired resistance to WZ4002. WZ4002-resistant cells were treated with WZ4002, U0126, or a combination of two drugs for 72 h. Cellular viability was determined by MTT assay.
